# Supplementary material for: Convergent causal mapping unravels distinct frontal networks for visuospatial selective attention
Source: Nat Commun. 2025 Dec 15;17:659. doi: 10.1038/s41467-025-67381-5 (PMC12816660; doi:10.1038/s41467-025-67381-5)
Supplement: Supplementary file 1 — Supplementary Information [file 41467_2025_67381_MOESM1_ESM.pdf]

Convergent causal mapping unravels distinct frontal networks for visuospatial selective attention

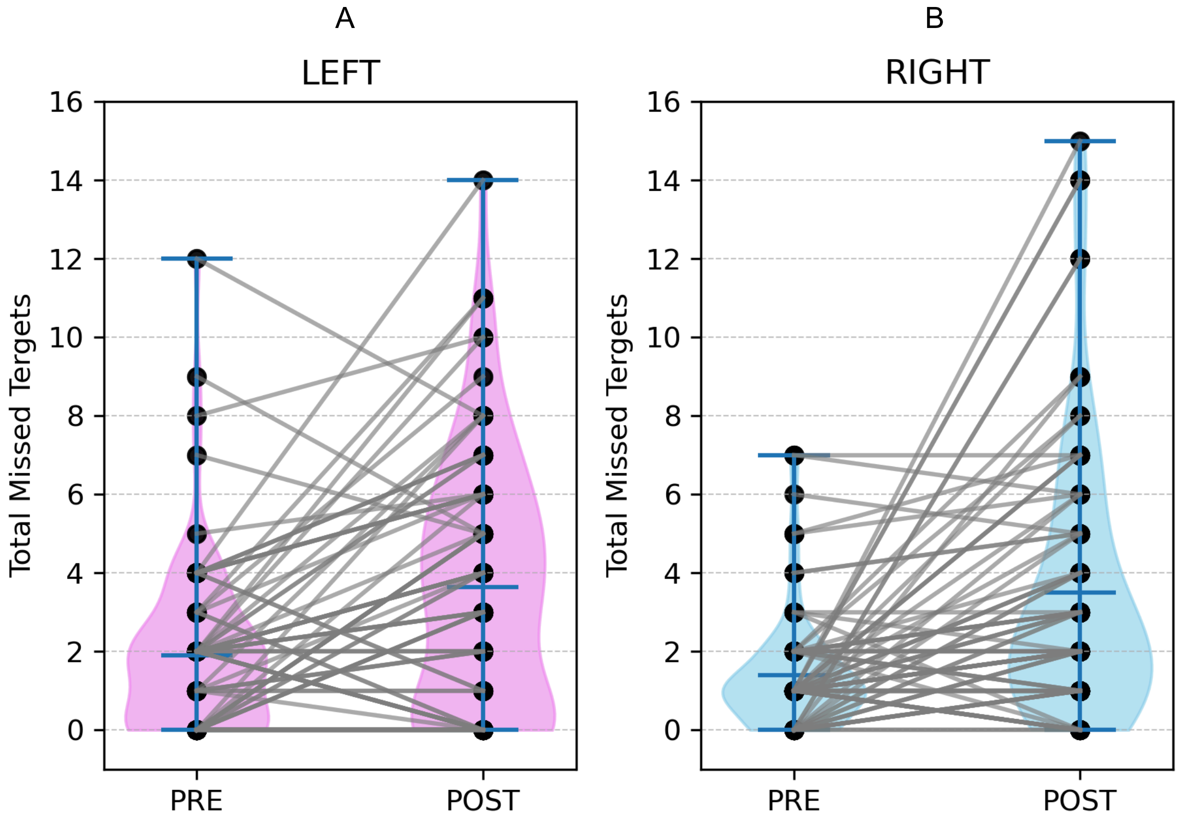

Supplementary Figure 1. Pre/Postoperative Bells Test total score by Hemisphere (retrospective group). Pre and post operative visuospatial attention performances (total missed targets) of retrospective sample for left (pink, panel A) and right (light blue, panel B) hemispheres (left: n=81; right: n=82). Individual data points are displayed as overlaid dots on the violin plots. A repeated-measures ANOVA revealed a significant increase in total score from pre-surgery (right hemisphere mean = 1.39(±1.50); left hemisphere mean = 1.9(±2.19)) to post-surgery (right hemisphere mean = 3.50(±3.46); left hemisphere mean = 3.64(±3.29)); F(1,161)=54.23, p<.001, η^2□=.252 95% CI [0.177, 0.331]) with no significant Time×Hemisphere interaction (F(1,161)=0.50, p=.481, η^2□=.003 95% CI [0.000, 0.041]). Wilcoxon signed-rank tests confirmed a statistically significant increase in total score following surgery in both left-hemisphere (n=81; Z=–4.35, p<.001, r_rb = 0.632, 95% CI [0.420, 0.819]) and right-hemisphere patients (n=82; Z=–5.25, p<.001 r_rb = 0.725, 95% CI [0.549, 0.867]), corroborating the ANOVA. Source data are provided as a Source Data file.

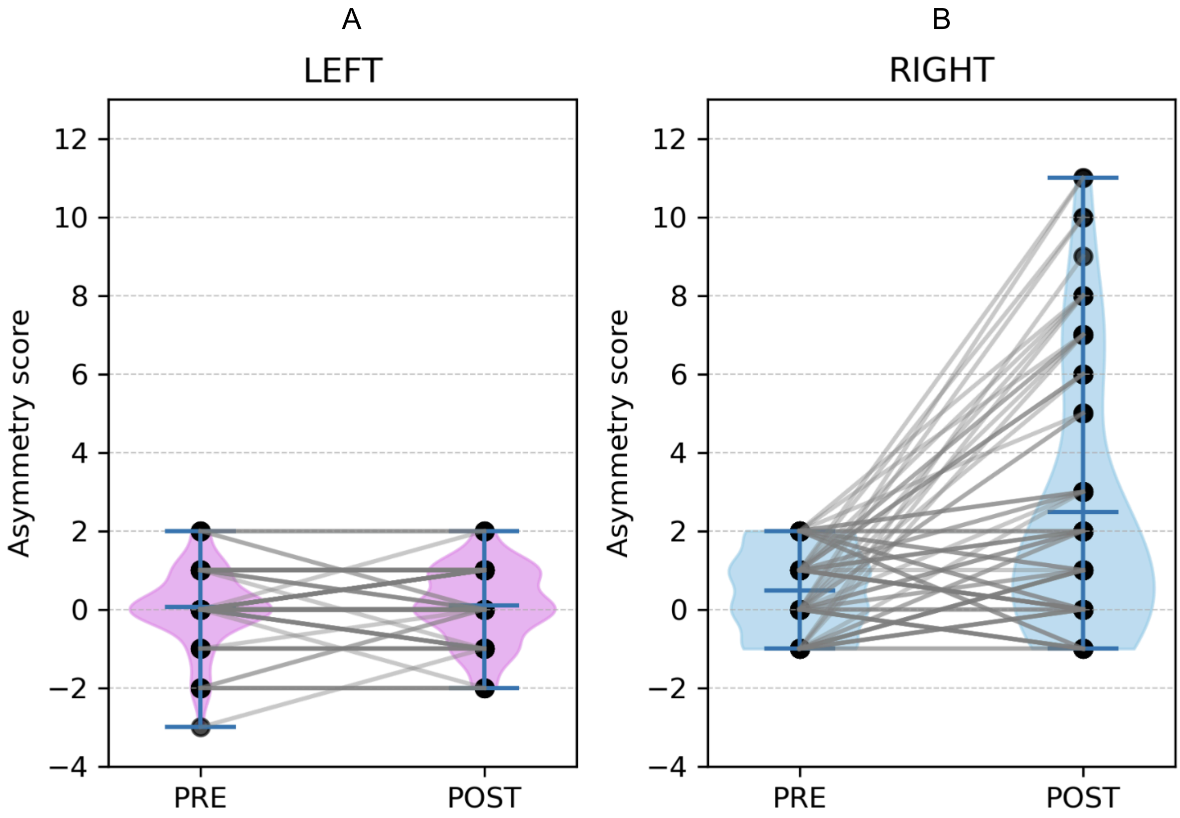

Supplementary Figure 2. Pre- and post-operative Bells Test asymmetry scores (retrospective study). The scores of the retrospective sample for left (pink, panel A) and right (light blue, panel B) hemispheres (left: n=81; right: n=82) are displayed. Individual data points are displayed as overlaid dots on the violin plots. A repeated-measures ANOVA revealed a significant Time×Hemisphere interaction ($F(1,161)=24.92$, $p<.001$, η^2□=.134, 95% CI [−0.274, 0.177]). Post-hoc tests showed left-hemisphere patients had no significant postoperative change in asymmetry score (mean pre 0.06±0.98, post 0.1±0.97; two sided $t(80)= -0.395$, $p=0.694$, Cohen's d=-0.044, 95% CI [−0.274, 0.177]), whereas patients with right lesions showed a significantly higher score (mean pre 0.49±1.08, post 2.48±3.29; two sided $t(81)=–5.272$, $p<0.001$, Cohen's d^z = −0.582, 95% CI [−0.765, −0.413]). Wilcoxon tests confirmed these findings: no change for left-hemisphere patients (n=81; Z=–0.37, p=.712, r_rb = 0.070, 95% CI [−0.303, 0.435]) but a significant postoperative increase for right-hemisphere patients (n=82; Z=–4.53, p<.001, r_rb = 0.604, 95% CI [0.399, 0.774]). Source data are provided as a Source Data file.

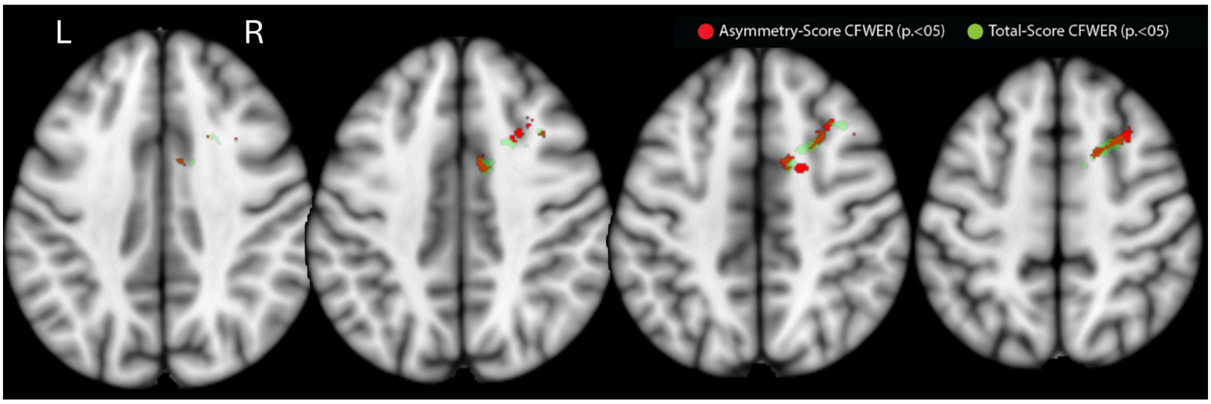

Supplementary Figure 3. **Asymmetry and total score significant SVR-LSM clusters overlap.** The cluster associated with the impairment in the asymmetry score of the Bell Test after correction for cluster level family-wise error is shown in red (CFWER, P = 0.05, v = 1). In green, the significant cluster associated with the total-score is displayed (CFWER, P = 0.05, v = 1). N=82 right hemisphere patients.

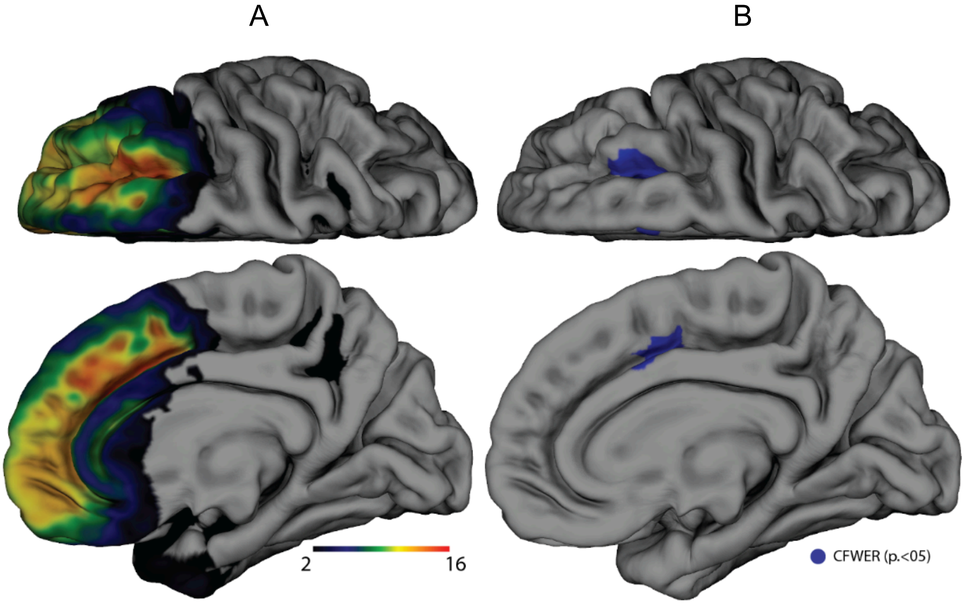

Supplementary Figure 4. Coherence of SVR-LSM findings with a clinical-categorical lesion analysis. A: Resection overlap map of the subgroup of patients (n=21) with a postoperative pathological asymmetry score. The colour bar indicates the number of overlapping resections. B: The significant cluster from the SVR-LSM analysis (CFWER, P = 0.05, v = 1) (n=82) is shown for comparison.

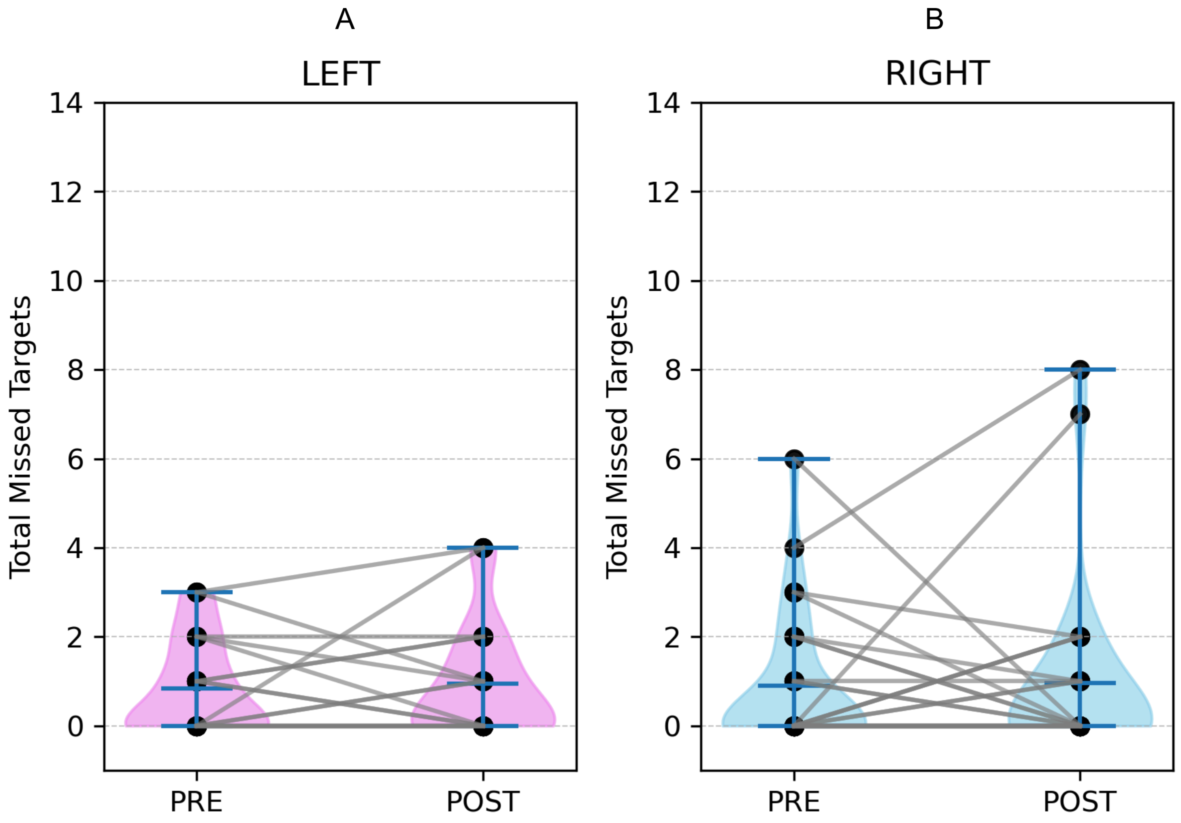

Supplementary Figure 5. Pre- and post-operative Bells Test total scores in the prospective study. Pre- and post-operative visuospatial attention performances of the prospective cohort for left (pink, panel A) and right (light blue, panel B) hemispheres (left: n=19; right: n=28). Individual data points are displayed as overlaid dots on the violin plots. Statistical analysis was performed using repeated-measures ANOVA. No significant main effect of Time was observed for total score ($F(1,45)=0.10$, $p=.76$, partial η^2□ = 0.000, 95% CI [0.000, 0.107]), and the Time × Hemisphere interaction was non-significant ($F(1,45)=0.003$, $p=.95$, η^2□ = 0.000, 95% CI [0.000, 0.107]). Source data are provided as a Source Data file.

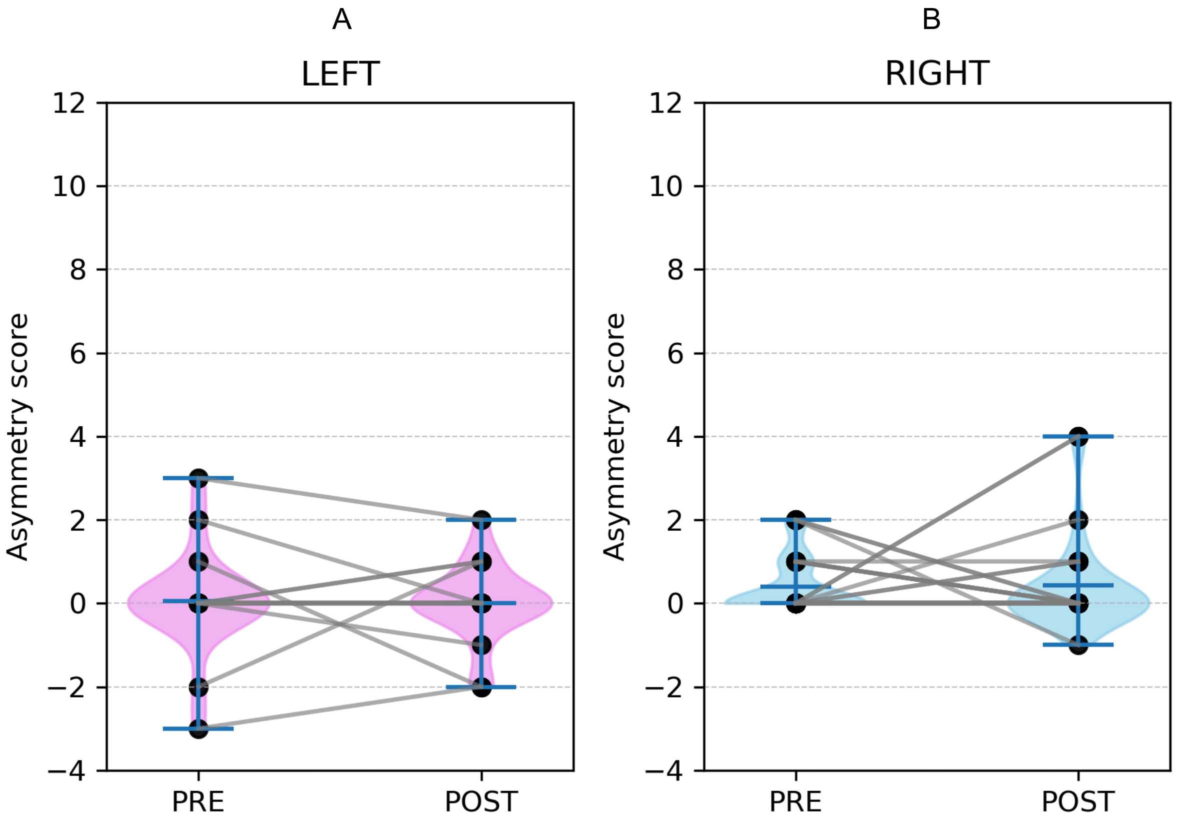

Supplementary Figure 6. Pre- and post-operative Bells Test asymmetry scores in the prospective study. Pre- and post-operative visuospatial attention asymmetry scores of the prospective cohort for left (pink, panel A) and right (light blue, panel B) hemispheres (left: n=19; right: n=28). Individual data points are displayed as overlaid dots on the violin plots. Statistical analysis was performed using repeated-measures ANOVA. No significant main effect of Time was observed ($F(1,45)=0.002$, $p=.967$, η^2□ = 0.000, 95% CI [0.000, 0.110]), and the Time × Hemisphere interaction was non-significant ($F(1,45)=0.046$, $p=.831$, η^2□ = 0.001, 95% CI [0.000, 0.110]). Source data are provided as a Source Data file.

Characteristic	Right Hemisphere (n=82)	Left Hemisphere (n=81)
**Age (years), mean ± SD**	38.55 ± 12.14	49.37 ± 12.52
**Education (years), mean ± SD**	14.22 ± 2.60	14.42 ± 3.03
**Tumor Grade, n (%)**		
Low-Grade	36 (44%)	33 (41%)
High-Grade	46 (56%)	48 (59%)
**Sex, n (%)**		
Female	39 (48%)	33 (41%)
Male	43 (52%)	48 (59%)
**Handedness, n (%)**		
Right-handed	79 (96%)	75 (93%)
Left-handed	3 (4%)	6 (7%)
**Bells Test - Total Score, mean ± SD**		
Pre-operative	1.39 ± 1.50	1.90 ± 2.19
Post-operative	3.50 ± 3.46	3.64 ± 3.29
**Bells Test - Asymmetry Score, mean ± SD**		
Pre-operative	0.49 ± 1.08	0.06 ± 0.98
Post-operative	2.48 ± 3.29	0.10 ± 0.97

Supplementary Table 1. Sociodemographic / clinical characteristics and average attentional scores of the patients (retrospective study).

Area	Z_Strength	Z_Degree	Z_Betweenness	Z_Composite	Frequency_Composite
R-6ma	8.57639215	4.672522584	8.840410574	7.363108436	95
R-i6-8	8.879815651	4.5322699	8.224290379	7.21212531	96
R-8Av	8.790855253	4.525480351	7.578237089	6.964857564	100
R-SCEF	4.418215324	4.387361526	8.837042352	5.880873067	97
R-Thalamus	3.452962865	2.921982863	7.880675254	4.751873661	99
R-SFL	3.728454208	4.50608164	2.925896527	3.720144125	98
R-6a	5.406150457	3.429841129	2.012318198	3.616103261	84
R-s6-8	3.011284072	2.894242706	0.892629342	2.26605204	55
R-8C	3.291940682	2.804814646	0.670224377	2.255659901	61
R-p24pr	1.661006295	2.205394462	1.759835535	1.875412097	52

Supplementary Table 2. Consensus Hubs of the Normative Visuospatial Neglect Network (n=100 HCP subjects). Graph theory metrics for the 10 consensus hubs identified from the normative connectome analysis. Hubs were defined as nodes with a composite hub z-score (integrating node strength, degree, and betweenness centrality) exceeding 1.5 in at least 50% of the 100 HCP subjects. The table reports the mean Z-scores for each centrality measure, the final composite hub score, and the number of subjects in whom the composite score was above the Z > 1.5 threshold. Hub labels correspond to the HCP-MMP1.0 atlas.

Characteristic	Right Hemisphere (n=28)	Left Hemisphere (n=19)
**Age (years), mean ± SD**	41.71 ± 10.05	43.11 ± 11.10
**Education (years), mean ± SD**	14.64 ± 2.71	15.05 ± 2.59
**Tumor Grade, n (%)**		
Low-Grade	20 (71%)	9 (47%)
High-Grade	8 (29%)	10 (53%)
**Sex, n (%)**		
Female	14 (50%)	11 (58%)
Male	14 (50%)	8 (42%)
**Handedness, n (%)**		
Right-handed	26 (93%)	18 (95%)
Left-handed	2 (7%)	1 (5%)
**Bells Test - Total Score, mean ± SD**		
Pre-operative	0.89 ± 1.52	0.84 ± 1.07
Post-operative	0.96 ± 1.99	0.95 ± 1.31
**Bells Test - Asymmetry Score, mean ± SD**		
Pre-operative	0.39 ± 0.69	0.05 ± 1.22
Post-operative	0.43 ± 1.14	0.00 ± 0.94

Supplementary Table 3. Sociodemographic / clinical characteristics and attentional scores of the patients (prospective study).

Supplementary methods

Intraoperative brain mapping and monitoring

All surgeries were performed in asleep-awake-asleep anaesthesia with the aid of brain mapping and monitoring techniques. The craniotomy exposed the tumour area and a limited portion of the surrounding cortex. For cortical and subcortical motor mapping, High frequency DES (HF-DES) was delivered using a constant current monopolar stimulator (straight tip, 1.5mm diameter, Inomed, with reference/ground on the skull overlying the central sulcus) in trains of 5 (To5) constant anodal current pulses (pulse duration: 5msec, interstimulus interval ISI: 3-4msec). To identify and preserve sites producing interferences on language, motor/praxis[1-6], visual[7] and executive functions[8-9] Low-Frequency DES was used, delivered by a 5 mm distance tip (60 Hz, pulse width = 0.5 ms, biphasic current, 1–4 s of stimulation) probe, in awake anaesthesia. The lowest current intensity applied to the ventral premotor cortex that interfered with language task was used for the cortical and subcortical mapping aimed at searching the tumour functional boundaries since the beginning of the resection. When the tumour was functionally disconnected, it was then removed in general anaesthesia under HF-DES motor mapping and brain monitoring techniques. In all patients, monitoring included simultaneous acquisition of continuous EEG, ECoG, free-running EMG, motor-evoked potentials (MEPs) and somatosensory-evoked potentials (SEPs). EEG (10/20 system) and ECoG (4/6-contact subdural grid over the precentral gyrus) were recorded to detect seizures, afterdischarges during stimulation and depth of anaesthesia to titrate the level of anaesthetics. The integrity of the descending motor pathways was monitored throughout the procedure by using the To5 monitoring technique (pulse duration 0.5–0.8 milliseconds; ISI 2–4 milliseconds, repetition rate 1-1.5 Hz) delivered to M1 to elicit MEPs, either by transcranial electrodes and direct cortical stimulation[10].

Supplementary References

1. Bello, L. et al. Tailoring neurophysiological strategies with clinical context enhances resection and safety and expands indications in gliomas involving motor pathways. Neuro-Oncol. 16, 1110–1128 (2014).

2. Fornia, L. et al. Direct electrical stimulation of premotor areas: different effects on hand muscle activity during object manipulation. Cereb. Cortex 30, 391–405 (2020).

3. Fornia, L. et al. The parietal architecture binding cognition to sensorimotor integration: a multimodal causal study. Brain 147, 297–310 (2024).

4. Rossi, M. et al. Assessment of the praxis circuit in glioma surgery to reduce the incidence of postoperative and long-term apraxia: a new intraoperative test. J. Neurosurg. 130, 17–27 (2019).

5. Rossi, M. et al. Clinical pearls and methods for intraoperative motor mapping. Neurosurgery 88, 457–467 (2021).

6. Viganò, L. et al. Stimulation of frontal pathways disrupts hand muscle control during object manipulation. Brain 145, 1535–1550 (2022).

7. Conti Nibali, M. et al. Preserving visual functions during gliomas resection: feasibility and efficacy of a novel intraoperative task for awake brain surgery. Front. Oncol. 10, 1485 (2020).

8. Puglisi, G. et al. Preserving executive functions in nondominant frontal lobe glioma surgery: an intraoperative tool. J. Neurosurg. 131, 474–480 (2018).

9. Puglisi, G. et al. Frontal pathways in cognitive control: direct evidence from intraoperative stimulation and diffusion tractography. Brain 142, 2451–2465 (2019).

10. Viganò, L. et al. Transcranial versus direct electrical stimulation for intraoperative motor-evoked potential monitoring: prognostic value comparison in asleep brain tumor surgery. Front. Oncol. 12, 963669 (2022).
