# Supplementary material for: Convergent causal mapping unravels distinct frontal networks for visuospatial selective attention
Source: Nat Commun. 2025 Dec 15;17:659. doi: 10.1038/s41467-025-67381-5 (PMC12816660; doi:10.1038/s41467-025-67381-5)
Supplement: Supplementary file 2 — Reporting Summary [file 41467_2025_67381_MOESM2_ESM.pdf]

nature portfolio

Corresponding author(s): Luca Viganò

Last updated by author(s): Nov 7, 2025

Reporting Summary

Nature Portfolio wishes to improve the reproducibility of the work that we publish. This form provides structure for consistency and transparency in reporting. For further information on Nature Portfolio policies, see our Editorial Policies and the Editorial Policy Checklist.

Statistics

For all statistical analyses, confirm that the following items are present in the figure legend, table legend, main text, or Methods section.

n/a	Confirmed	
☐	☒	The exact sample size (*n*) for each experimental group/condition, given as a discrete number and unit of measurement
☐	☒	A statement on whether measurements were taken from distinct samples or whether the same sample was measured repeatedly
☐	☒	The statistical test(s) used AND whether they are one- or two-sided<br>*Only common tests should be described solely by name; describe more complex techniques in the Methods section.*
☐	☒	A description of all covariates tested
☐	☒	A description of any assumptions or corrections, such as tests of normality and adjustment for multiple comparisons
☐	☒	A full description of the statistical parameters including central tendency (e.g. means) or other basic estimates (e.g. regression coefficient) AND variation (e.g. standard deviation) or associated estimates of uncertainty (e.g. confidence intervals)
☐	☒	For null hypothesis testing, the test statistic (e.g. *F*, *t*, *r*) with confidence intervals, effect sizes, degrees of freedom and *P* value noted<br>*Give P values as exact values whenever suitable.*
☒	☐	For Bayesian analysis, information on the choice of priors and Markov chain Monte Carlo settings
☒	☐	For hierarchical and complex designs, identification of the appropriate level for tests and full reporting of outcomes
☐	☒	Estimates of effect sizes (e.g. Cohen's *d*, Pearson's *r*), indicating how they were calculated

Our web collection on statistics for biologists contains articles on many of the points above.

Software and code

Policy information about availability of computer code

Data collection	No specifc codes were used for data collection
Data analysis	- SPM12 (clinical toolbox) was used to perform the enantiomorphic normalization of T1w and resection cavities (https://www.fil.ion.ucl.ac.uk/spm/software/spm12/)<br>- ITK-SNAP was used to segment the resection cavities (Yushkevich PA et al. 2016)<br>- SVR-LSM was performed using a MATLAB toolbox released by DeMarco and Turkeltaub (DeMarco & Turkeltaub, 2018)<br>- Lesion Quantification Toolkit was used to compute the parcel-level lesion load (Griffis et al. 2021)<br>- MRTrix3 was used for diffusion MRI processing, constrained spherical deconvolution, probabilistic tractography, connectome construction and TDI imaging (https://www.mrtrix.org/).<br>- ANTs (Advanced Normalization Tools) was used for image registration (https://github.com/ANTsX/ANTs)<br>- App-ants-mni via brainlife.io (Avesani et al. 2019) was used to for non-linear registration of streamlines (.tck) to MNI152.<br>- FreeSurfer was used for cortical and subcortical parcellation (https://surfer.nmr.mgh.harvard.edu/)<br>- The Brain Connectivity Toolbox (BCT) was used for graph theory analyses in MATLAB (https://sites.google.com/site/bctnet/)<br>- Peaglet sofware (Bellacicca et al., 2024) was used to compute probability kernel density estimation (PDE analysis) of intraoperative eloquent sites<br>- IBM SPSS 24.0 software was used for statistical analyses

For manuscripts utilizing custom algorithms or software that are central to the research but not yet described in published literature, software must be made available to editors and reviewers. We strongly encourage code deposition in a community repository (e.g. GitHub). See the Nature Portfolio guidelines for submitting code & software for further information.

Data

Policy information about availability of data

All manuscripts must include a data availability statement. This statement should provide the following information, where applicable:

- Accession codes, unique identifiers, or web links for publicly available datasets
- A description of any restrictions on data availability
- For clinical datasets or third party data, please ensure that the statement adheres to our policy

The group-level neuroimaging results generated in this study (SVR-LSM significative cluster; population-average TDI map; PDEs of intraoperative iVSAT interferences; average TDI maps associated with iVSAT errors) have been deposited in the Zenodo repository under accession code [https://doi.org/10.5281/zenodo.17544485]. The de-identified minimum dataset necessary to reproduce the study's findings, including individual-level demographic, clinical, processed intraoperative and neuroimaging data, is available under restricted access to protect patient confidentiality, as stipulated by the ethical approval for this study. Access is subject to approval by the IRCCS Ospedale Galeazzi-Sant'Ambrogio and will require the signing of a Data Use Agreement (DUA). Requests for access should be directed to the corresponding author. The raw patient neuroimaging data are protected and are not available due to data privacy regulations. The source data underlying the figures, including fully anonymized behavioural scores and disconnection metrics, are provided as a Source Data file with this paper. The Human Connectome Project (HCP) '100 unrelated subjects' dataset used in this study is publicly available from the ConnectomeDB database (db.humanconnectome.org).

Research involving human participants, their data, or biological material

Policy information about studies with human participants or human data. See also policy information about sex, gender (identity/presentation), and sexual orientation and race, ethnicity and racism.

Reporting on sex and gender	Sex of patients is reported in the Supplementary Information. Analyses were not performed separately for this variable, as the study is not designed to investigate sex-based differences.
Reporting on race, ethnicity, or other socially relevant groupings	Not applicable
Population characteristics	Basic demographic and tumour-related variables are provided in the supplementary materials. For SVR-LSM analysis, age, education level, and tumour grade were used as covariates.
Recruitment	- Retrospective study: all patients with brain tumour who underwent an aslee-awake-asleep procedure for brain tumour removal were included if meeting the following inclusion criteria: 1) absence of pre- and postoperative visual, motor or comprehension deficits; 3) absence of previous neurosurgical procedures or adjuvant treatments; 4) available neuropsychological preoperative and postoperative scores; 5) available preoperative and postoperative MRI.<br>- Prospective study: The inclusion criteria were the same adopted in the retrospective study, except for one condition: patients presenting with preoperative visuospatial attention deficits were excluded due to the potential impact of such deficits on the intraoperative administration and reliability of intraoperative attention assessment.<br>All patients meeting those criteria candidate to an asleep-awake-asleep procedure for brain tumour removal between 07/03/2024 and 16/09/2024 were included. These patients performed the intraoperative visuospatial selective attention task (iVSAT).<br><br>A potential limitation to generalizability of results is that tumour localization cannot be established a priori. In our sample, the parietal lobe was less frequently affected, possibly producing an underestimation of the role of this region in visuospatial selective attention (see Discussion).
Ethics oversight	Patients gave formal consent to the procedures, and the study was approved by the local ethical committee<br><br>- Retrospective study: data was used under approval of the Comitato Etico Territoriale Lombardia 1 (L2093)<br>- Prospective study: data were used under approval of the Comitato Etico Territoriale Lombardia 1 (study AIRC - 17482)

Note that full information on the approval of the study protocol must also be provided in the manuscript.

Field-specific reporting

Please select the one below that is the best fit for your research. If you are not sure, read the appropriate sections before making your selection.

☒ Life sciences ☐ Behavioural & social sciences ☐ Ecological, evolutionary & environmental sciences

For a reference copy of the document with all sections, see nature.com/documents/nr-reporting-summary-flat.pdf

Life sciences study design

All studies must disclose on these points even when the disclosure is negative.

Sample size	- Retrospective study: no sample size calculation was performed. Brain tumour represents a rare CNS disease. This study benefits from a large

	dataset of brain tumour patients with pre- and postoperative neuropsychological data. We evaluated the prediction accuracy and reproducibility of the SVR-LSM results, all falling within the range considered as reliable (Fornia et al., 2024; Königsberg et al., 514 2021; Mandal et al., 2020).<br>- Prospective study: no sample size calculation was assessed, as no statistical differences or associations across groups were performed. To the best of our knowledge, this study has the largest sample size ever published with intraoperative testing of visuospatial selective attention.
Data exclusions	- Retrospective study: 171 patients were considered in the retrospective cohort. Of these, 8 patients were excluded due to visual deficits: 2 had hemianopia, and 6 had quadrantanopia. - Prospective study: Of the 50 patients considered for enrolment, 47 patients (28 right hemisphere) undergoing brain tumour resection in awake anaesthesia met the inclusion criteria. One patient was excluded due to hemianopia, and two were excluded for visuospatial attention deficits.
Replication	- Retrospective study: for SVR-LSM we assessed the prediction accuracy and reproducibility of the SVR-LSM results. Optimization of hyperparameters was performed via resubstitution loss and Bayesian optimization with 200 iterations and 5-fold cross validation.<br>- Prospective study: 1) Within subject: A stimulation site was considered positive for interference during the iVSAT when an error, i.e. a missed target, occurred in three non-consecutive stimulation trials. 2) Across subjects: a probability density estimation analysis (Bellacicca et al. 2024) was used to highlight regions associated with specific intraoperative responses. This revealed replicability of intraoperative results across patients.
Randomization	Our prospective study did not include a control group; therefore, randomization was not applicable
Blinding	Our prospective study did not include a control group; therefore, blinding was not applicable

Reporting for specific materials, systems and methods

We require information from authors about some types of materials, experimental systems and methods used in many studies. Here, indicate whether each material, system or method listed is relevant to your study. If you are not sure if a list item applies to your research, read the appropriate section before selecting a response.

Materials & experimental systems

n/a	Involved in the study	
☒	☐	Antibodies
☒	☐	Eukaryotic cell lines
☒	☐	Palaeontology and archaeology
☒	☐	Animals and other organisms
☒	☐	Clinical data
☒	☐	Dual use research of concern
☒	☐	Plants

Methods

n/a	Involved in the study	
☒	☐	ChIP-seq
☒	☐	Flow cytometry
☐	☒	MRI-based neuroimaging

Plants

Seed stocks	N/A
Novel plant genotypes	N/A
Authentication	N/A

Magnetic resonance imaging

Experimental design

Design type	- Support vector regression lesion-symptom mapping (SVR-LSM)<br>- Structural connectome (mrtrix)<br>- Track density imaging (TDI) (mrtrix)<br>- Tractography in single patients: 1) Retrospective study: 1.11 Validation of consensus edge disconnection against postoperative deficits. 2) Prospective study: 2.5 Identification of stimulated white matter pathways (TDI imaging).
Design specifications	Not applicable
Behavioral performance measures	- For SVR-LSM we used as dependent variable the Bell Test's Δ total score (i.e. difference between the number of

omitted targets between pre- and post-operative assessment at 1 month) and the Δ asymmetry score (i.e. difference between the pre- and 1-month post-operative asymmetry score).
- Tractography in single patients: 1) Retrospective study: percentage of edge disconnection (FBC) was correlated with the Bells Test's Δ asymmetry score. 2) Prospective study: analysis of iVSAT omitted targets and lateralization index.

Acquisition

Imaging type(s)

Structural (resection cavities drawn from postoperative T1-MPRAGE)
Diffusion tractography

Field strength

3T

Sequence & imaging parameters

A post-contrast gadolinium T1-MPRAGE sequence was performed using the following parameters echo time: 2.75 ms, repetition time: 1600 ms, flip angle 9°, inversion time 900 ms; 176 slices; isotropic voxel size of 1 mm.

Area of acquisition

Whole-brain

Diffusion MRI ☒ Used ☐ Not used

Parameters

HARDI single-shell dataset: a spin-echo, single-shot echo-planar imaging diffusion sequence was acquired. 73 volumes were collected: 64 diffusion-weighted volumes at a b-value of 2000 s/mm^2 and 9 interleaved non-diffusion-weighted (b = 0 s/mm^2) volumes in the AP phase encoding direction. Repetition time = 16.9 s; echo time = 96 ms. A GRAPPA acceleration (parallel reduction factor = 2) was employed. Data were acquired with an isotropic voxel size of 2 mm^3 (matrix = 128 × 128, 64 slices, slice thickness = 2 mm) and a flip angle of 90°.

Preprocessing

Preprocessing software

T1-MPRAGE: SPM12 clinical toolbox.
MRTrix3 was used for diffusion MRI processing, constrained spherical deconvolution, probabilistic tractography, connectome construction and TDI imaging (https://www.mrtrix.org/).
ANTs (Advanced Normalization Tools) was used for image registration (https://github.com/ANTsX/ANTs).
FreeSurfer was used for cortical and subcortical parcellation (https://surfer.nmr.mgh.harvard.edu/).

Normalization

T1-MPRAGE: Enantiomorphic normalization using SPM12 Clinical Toolbox.
ANTs (Advanced Normalization Tools) was used for image registration of tractograms to MNI and of postoperative T1w/resection cavities to dMRI (https://github.com/ANTsX/ANTs; “app-ants-mni”; Avesani et al. 2019).
App-ants-mni via brainlife.io (Avesani et al. 2019) was used to for non-linear registration of streamlines (.tck) to MNI152.

Normalization template

MNI152

Noise and artifact removal

For dMRI:
Raw diffusion-weighted images were denoised using Marchenko–Pastur PCA (Veraart et al 2016a; 2016b). Images were corrected for Gibbs ringing artifacts (Kellner et al. 2016). Susceptibility and eddy current distortions were removed using the Synb0-DisCo “synthetic b0” approach (Schilling et al. 2019). B1 bias-field correction was performed using the ANTs N4 algorithm (Tustison et al. 2010).

Volume censoring

Not applicable

Statistical modeling & inference

Model type and settings

- Univariate tests were used to assess the task performance at the different time points (preoperative and postoperative). Non-parametric wilcoxon test was used for non-normal distribution data.
- Multivariate support vector regression analysis for LSM
- Bivariate tests for correlation between percentage of edge's disconnection and Bells Test's Δ asymmetry score.

Effect(s) tested

- The Bells Test's total score and asymmetry score at the different time points (preoperative and postoperative) were compared considering the hemisphere as between factor by using a two-way ANOVA and paired samples t-test for each score.
- Association between lesion location and behavioral outcome (SVR-LSM).
- Correlation between percentage of disconnected edges and Bells Test's Δ asymmetry score.

Specify type of analysis: ☐ Whole brain ☐ ROI-based ☒ Both

Anatomical location(s)

First, we performed whole-brain probabilistic tractography for each HCP unrelated subject and patient cohort. In the HCP normative cohort, the significant SVR-LSM cluster was used as an ROI to filter the whole-brain tractograms, isolating the specific sub-network of interest. From these filtered streamlines, we constructed quantitative, region-to-region structural connectomes (379x379 matrices) weighted by Fibre Bundle Capacity. Graph theory analysis was then applied to these connectome matrices to identify the network's "consensus hubs" and "consensus edges." Furthermore, the filtered HCP tractograms were used to generate a population-average track-density image (TDI) to visualize the anatomical substrate of this normative network. Finally, on patient-specific data, we used resection cavities as exclusion ROIs to

correlate the percentage of an edge's disconnection with postoperative scores. Intraoperative stimulation sites were used as inclusion ROIs to visualize functionally-defined average TDIs.

Statistic type for inference<br>(See Eklund et al. 2016)	SVR-LSM: A voxelwise thresholding was applied after generating SVR-β maps with 5000 permutations (P<0.005)
Correction	SVR-LSM: To correct for multiple comparisons, a cluster-level family-wise error correction was applied (CFWER, P = 0.05, v = 1).<br>- Correlation between percentage of disconnected edges and Bells Test's Δ asymmetry score: a Bonferroni correction was applied.

Models & analysis

n/a	Involved in the study
☒	☐ Functional and/or effective connectivity
☐	☒ Graph analysis
☐	☒ Multivariate modeling or predictive analysis

Graph analysis	Graph Construction: 379×379 weighted, unthresholded FBC matrices per subject, filtered via SIFT2 and restricted to streamlines passing through the SVR-LSM cluster. Node-Level Dependent Variables: node strength, node degree, and betweenness centrality computed for each HCP subject. Composite Hub Score: mean of Z-scored node metrics per subject; “consensus hubs” are nodes with Z > 1.5 in ≥50% of subjects. Edge-Level Analysis: for each consensus hub, edges with Z-cored FBC > 1.5 in ≥50% of subjects defined as “consensus top edges.”
Multivariate modeling and predictive analysis	A support vector regression lesion symptom mapping (SVR-LSM) was performed to assess the association between post-operative visuospatial selective attention deficit and specific brain areas. Optimization of hyperparameters was performed via resubstitution loss and Bayesian optimization with 200 iterations and 5-fold cross validation. In addition, the range for the optimised parameters was set following the range of C and Gamma suggested by (Zhang et al., 2014) and more recently adopted in different studies (Fornia et al., 2024; Wiesen et al., 2019). C range = 1–80, Gamma equivalent Sigma range = 0.1–30. A default Epsilon range was set.
